# Management of Atrial Fibrillation in Critically Ill Patients

**DOI:** 10.1155/2014/840615

**Published:** 2014-01-16

**Authors:** Mattia Arrigo, Dominique Bettex, Alain Rudiger

**Affiliations:** Cardiosurgical Intensive Care Unit, Institute of Anesthesiology, University Hospital Zurich, Raemistraße 100, 8091 Zurich, Switzerland

## Abstract

Atrial fibrillation (AF) is common in ICU patients and is associated with a two- to fivefold increase in mortality. This paper provides a reappraisal of the management of AF with a special focus on critically ill patients with haemodynamic instability. AF can cause hypotension and heart failure with subsequent organ dysfunction. The underlying mechanisms are the loss of atrial contraction and the high ventricular rate. In unstable patients, sinus rhythm must be rapidly restored by synchronised electrical cardioversion (ECV). If pharmacological treatment is indicated, clinicians can choose between the rate control and the rhythm control strategy. The optimal substance should be selected depending on its potential adverse effects. A beta-1 antagonist with a very short half-life (e.g., esmolol) is an advantage for ICU patients because the effect of beta-blockade on cardiovascular stability is unpredictable in those patients. Amiodarone is commonly used in the ICU setting but has potentially severe cardiac and noncardiac side effects. Digoxin controls the ventricular response at rest, but its benefit decreases in the presence of adrenergic stress. Vernakalant converts new-onset AF to sinus rhythm in approximately 50% of patients, but data on its efficacy and safety in critically ill patients are lacking.

## 1. Introduction

Atrial fibrillation (AF) is the most common arrhythmia in patients hospitalised in intensive care units (ICUs) and is associated with increased morbidity and mortality [1–6]. In light of the improved understanding of the underlying pathophysiology, novel therapeutic options, and recently published guidelines for AF, this paper provides a reappraisal of the topic with a special focus on the management of AF in critically ill patients with haemodynamic instability.

## 2. Materials and Methods

A search of the PubMed database and a review of bibliographies from selected articles was performed to identify original data relating to this topic. Articles were scrutinised regarding their study design, population evaluated, interventions, outcomes, and limitations. A special focus was on the literature available from critically ill patients. However, if such information was lacking, references from non-ICU patients were included in this narrative review. When evidence-based recommendations were not available at all personal recommendations were incorporated in this report (and highlighted accordingly) to assist the clinicians in the management of critically ill patients with AF.

## 3. Results and Discussion

### 3.1. Definition and Clinical Manifestation

AF is a supraventricular arrhythmia characterized by disorganized atrial depolarisations without effective atrial contractions. If AF terminates spontaneously, it is defined as *paroxysmal*. When AF is sustained beyond seven days or is terminated with electrical or pharmacological cardioversion it is defined as *persistent*. If a conversion in sinus rhythm cannot be achieved, AF is defined as *permanent* [5].

In critically ill patients, untreated AF can cause hypotension (mean arterial pressure < 65 mmHg), myocardial ischemia, and heart failure (pulmonary edema, cardiogenic shock) with subsequent tissue hypoxia (SvO2 < 65%, lactate > 2.0 mmol/l) and organ dysfunction (encephalopathy, acute kidney injury with urine output < 0.5 mL/kg/h and liver dysfunction). The underlying mechanisms of these complications are the loss of atrial contraction and the high ventricular rate, which both impair the ventricular filling. The loss of the atrial kick is particularly detrimental in patients with diastolic dysfunction, such as left ventricular hypertrophy of any cause. Left atrial pressure increases, causing pulmonary venous hypertension and subsequent pulmonary edema with dyspnea. When stroke volume deteriorates, cardiogenic shock develops [7]. In addition, the high heart rate and the secondary elevation of end-diastolic ventricular pressure increase the myocardial oxygen demands, precipitating acute myocardial ischemia. Uncontrolled tachycardia for the duration of days to weeks may cause tachycardia-induced myocardial dysfunction (tachycardiomyopathy) leading to severe systolic heart failure, which is potentially reversible after appropriate treatment [8, 9].

### 3.2. Diagnostic Evaluation

AF is diagnosed by a 12-lead electrocardiogram (ECG), typically when a lack of *P*-waves, high-frequency fibrillation waves at rates of 350–600/min, and an irregular ventricular response (*absolute arrhythmia*) are observed. The ventricular rate in untreated patients with normal atrioventricular conduction is typically between 100 and 160 bpm, but normo- and bradycardic ventricular response rates are possible. The initial ECG may provide important additional information on myocardial ischemia, left-ventricular hypertrophy, or conduction disorders. When the differentiation of narrow-complex tachycardia is challenging, 6 mg of adenosine pushed intravenously can terminate a reentry tachycardia or unmask atrial flutter and AF [10]. Of note, adenosine can precipitate ventricular tachycardia in preexcitation syndromes (e.g. Wolff-Parkinson-White) by rapid anterograde conduction of AF via the accessory pathway [11]. After cardiac surgery, an atrial lead ECG from the pacemaker wire can be helpful. The evaluation of symptoms and haemodynamic consequences is the next step [1]. If AF is accompanied by acute chest pain, dyspnea, arterial hypotension, and/or cardiogenic shock, immediate action is required (see below). Transthoracic echocardiography, chest radiography, and electrolyte and serologic tests for thyroid function are required to identify the underlying cause of the AF [12]. In cardiac surgery patients, transthoracic or transesophageal echocardiography may be necessary to rule out pericardial effusion, a common trigger for AF in the early postoperative phase.

### 3.3. Epidemiology

Advanced age is the biggest risk factors for developing AF. The incidence and prevalence rises with age (>60 years: 1%; >80 years: 5–15%) [13–18]. AF occurs in patients with cardiac disorders (hypertensive heart disease, coronary artery disease, valvular heart disease, pericarditis, congenital heart disease and acquired cardiomyopathies) as well as in patients with no apparent cardiac abnormalities (lone AF) [5]. Many noncardiac diseases (thyroid disorders, pulmonary diseases, and alcohol overconsumption) are also associated with AF [19, 20]. Acute illness and surgery are associated with increased rates of AF. The incidence of new-onset AF in critically ill patients is 6–20% [21–23]. In the subgroup of patients with sepsis, the incidence of new-onset AF correlates with the severity of sepsis; up to half of the patients with septic shock experience new-onset AF [24]. In cases with acute coronary artery disease, AF occurs in 6–21% of patients [25]. The highest incidence is observed in patients after open heart surgery, in particular mitral valve surgery and coronary artery bypass graft surgery, with documented rates reaching 30–40% [26, 27]. The peak incidence of AF occurs during the first 2–4 days after cardiac surgery [26]. Overall, AF is associated with cardioembolic events and heart failure, longer hospital stays, and reduced quality of life as well as a two- to fivefold increased mortality [21–23, 26, 28].

### 3.4. Underlying Mechanisms

The complex pathophysiological mechanisms of AF have been reviewed extensively [1–6, 29]. To better understand the various treatment options, some basic elements of AF are summarised below. Reentry of excitation wavefronts has long been considered the main mechanism of AF. However, intensive research during the recent decades has revealed an interaction between the initiation triggers and maintenance factors of AF.  Table 1 summarises the promoters of AF and what specific treatments, if any, they are amenable to [5, 26, 29–32]. Clearly, these promoters of AF are different in critically ill patients compared to outpatients. Any heart disease or cardiac surgery involving sutures on the atria can induce structural remodelling of the atria, which results in inflammation, myocyte alteration, and tissue fibrosis, all of which promote AF. A few minutes after the onset of AF, an electrical remodelling process involving ion channel function and intracellular calcium homeostasis is stimulated, leading to a shortening of the refractory periods of atrial cardiomyocytes and contributing to the persistence of AF [7, 33]. Within days, alterations to the intracellular calcium homeostasis cause contractile remodelling, dysfunction, and further dilatation of the atria [8, 9, 33]. Increased sympathetic tone and systemic inflammation also play a central role in maintaining AF [10, 34]. Inflammation may lead to atrial myocarditis with subsequent electrical and structural atrial changes, resulting in the initiation and maintenance of AF [11, 35]. Because AF triggers AF, paroxysmal AF might progress to persistent and permanent AF [1, 36]. Finally, an origin of AF has been localised in the myocardial sleeves of the pulmonary veins, opening the door to new ablation techniques [12, 37–41]. However, the complex mechanisms leading to this ectopic activity with bursts of rapid discharge are not yet fully understood.

### 3.5. Management of Patients with Haemodynamic Instability

The initial management of patients with haemodynamic instability includes the restoration of an adequate perfusion pressure with, depending upon the aetiology of AF, administration of fluids, vasopressors, and/or inotropes. Special attention should be addressed to sedation and analgesia, which ensure patient comfort and reduce the incidence of harmful sympathetic activation, and a sufficient oxygen supply of the myocardium must be guaranteed.

#### 3.5.1. Electrical Cardioversion (ECV)

**Figure 1:**
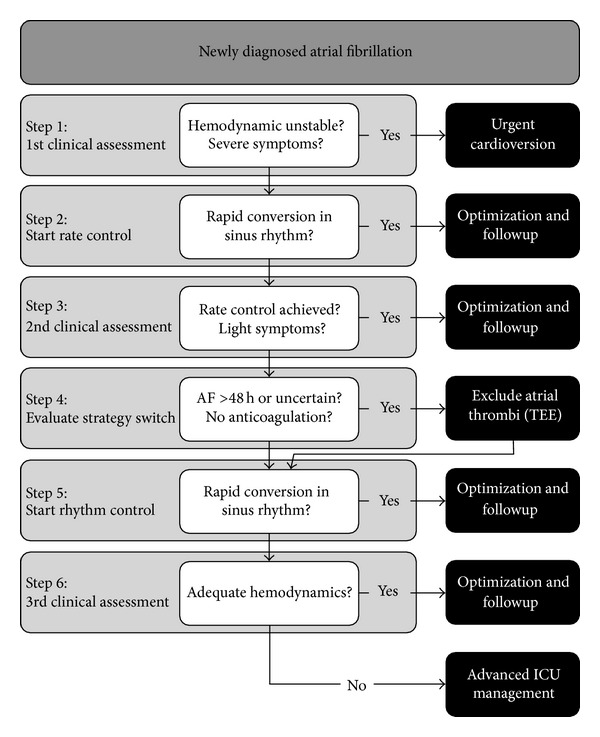
Management algorithm. Legend: ICU intensive care unit. Algorithm modified from [2, 4].

In patients with acute chest pain, dyspnea, or haemodynamic instability, the sinus rhythm must be rapidly restored by synchronised ECV (Figure 1). Compared to the success rate of 90% in outpatients [13–18, 42], the conversion rate is much lower in critically ill patients undergoing urgent cardioversion, with published success rates as low as 30% [5, 43–46]. Pretreatment with antiarrhythmic drugs facilitates ECV and reduces immediate recurrences [19, 20, 47, 48]. Chest wall impedance, left atrial size, and duration of AF are inversely related to success rate. Prior to ECV, patients should receive sedation and analgesia. Endotracheal intubation is required in patients at risk of aspiration. Anterior-posterior electrode positioning and biphasic waveforms provide higher success rates than lateral electrode positioning and monophasic waveforms [21–23, 42]. In postoperative cardiosurgical patients, for whom impedance is high and electrodes are often placed unfavourably due to wound dressing and chest tubes, we recommend a single shock of 200 Joules to increase the success rate [24, 49]. A previous study demonstrated that a high initial energy reduces the incidence of tachyarrhythmic complications [25, 50]. Particular care must be taken to preserve the wound dressings and to avoid the nipples. If repeated ECV is applied, the synchronisation mode has to be switched on before every use, as this mode usually switches off after every discharge to allow immediate defibrillation if necessary. In patients with pacemakers or internal cardioverter/defibrillators (ICD), internal overdrive pacing and/or cardioversion may be attempted by the cardiologists to restore the sinus rhythm. If this is not possible, the external electrodes should be placed at least 8 cm from the aggregate. After cardioversion, the device should be checked to ensure normal function.

In patients with life-threatening symptoms, ECV is indicated even if the presence of an atrial thrombus cannot be excluded. In stable patients with AF lasting more than 48 h, a transesophageal echocardiography to exclude an atrial thrombus is recommended [26, 27, 51]. Alternatively an adequate anticoagulation regimen of 3 weeks before cardioversion is recommended [6]. After successful cardioversion the anticoagulation should be continued for at least 4 weeks to prevent cardioembolic complications due to atrial stunning [6, 26]. If ECV was not successful, pharmacological treatment is indicated as described below. Similarly, an antiarrhythmic treatment is usually required temporarily to maintain sinus rhythm after successful ECV.

### 3.6. Management of Hemodynamic Stable Patients

#### 3.6.1. Rate versus Rhythm Control

Clinicians can choose between a rate control and a rhythm control strategy. The rate control approach tolerates AF but controls the ventricular response rate to improve the ventricular filling and avoid a tachycardiomyopathy. It is the treatment of choice in patients with permanent AF or in oligosymptomatic patients (Figure 1). Rate control can be accomplished with beta-blockers, calcium channel blockers (diltiazem, verapamil), digoxin, or amiodarone. 24 h telemetry should confirm that the target heart rate of less than 110 bpm at rest has been achieved [21–23, 26, 28, 52]. Some patients may experience clinical improvement only after the restoration of sinus rhythm (rhythm control strategy), which can be achieved by ECV and/or drugs (see Figure 1). However, several trials in outpatients failed to show a benefit of this strategy compared to rate control only [53, 54] even in patients with congestive heart failure [55]. The lack of a survival benefit in the rhythm control arm was probably caused by the inefficacy of current antiarrhythmic drugs and their adverse effects.

#### 3.6.2. Pharmacological Options

A multitude of substances are licensed for the pharmacologic treatment of AF, but only a few are indicated in the ICU setting (Table 2). Because the literature does not provide conclusive results on the optimal pharmacologic treatment of AF for ICU patients, clinicians should choose the optimal substance depending on its potential adverse effects [56]. Before starting an antiarrhythmic treatment, clinicians should optimise all concurring factors [56–58]: electrolyte derangements (potassium, magnesium) should be corrected to upper-normal levels. Particularly magnesium is an effective, cheap and well-tolerated treatment option for AF [3, 59–63].

We recommend to start with substances with a low risk profile and short half-life, such as betablockers (see below), and to escalate to other substance classes such as amiodarone only in cases of contraindications or inefficacy of the initial treatment. Generally, intravenous substances are preferred because of their faster onset and more reliable action.

Selective beta-1 receptor antagonists have negative chronotropic, dromotropic, and bathmotropic effects, slowing heart rate, delaying conduction in the atrioventricular node, and reducing myocardial excitability, respectively. Betablockers are therefore the initial treatment of choice for a rate control strategy. Adverse effects include the negative inotrope activity on the myocardium as well as vasodilatation [64] that can potentially worsen haemodynamics. Hence, a drug with a short half-life is recommended for ICU patients, for whom the effect of beta-blockade on cardiovascular stability is unpredictable. Our choice is esmolol which is eliminated by unspecific esterases and hydrolases resulting in a very short half-life of 7–10 minutes [65]. When esmolol treatment is initiated, we typically repeat intravenous esmolol injections of 10–20 mg to reach a dose of 1 mg/kg within a few minutes to assess its haemodynamic effects. If the mean arterial pressure remains above 60 mmHg, a continuous infusion is started at a rate of 0.05 mg/kg/min and is further increased in 30-minute intervals according to clinical needs. In patients with oral beta-blockers, therapy should be continued as it significantly reduces the risk of AF up to 40%, particularly in the postoperative phase [1, 60, 66–68].

Amiodarone is commonly used in the ICU setting for the treatment of AF. First of all, it has less negative inotropic effects compared to beta-blockers and calcium channel blockers [69]. Secondly, amiodarone is safer for patients with structural heart disease compared to class Ic antiarrhythmic agents, such as flecainide [3]. Amiodarone is a multichannel blocker with inhibiting effects on adrenergic receptors and potassium and calcium channels. It is a highly lipophilic substance with a very large distribution volume and an extremely long half-life [43, 45]. While a single dose of 150–300 mg of amiodarone is enough to achieve pharmacological conversion to sinus rhythm in some patients, the majority of patients require long-term therapy. Therefore, a loading dose of 0.1 g/kg is required in the first 7–10 days, which can be administered intravenously or orally. Thereafter, a daily oral maintenance dose of 200 mg is recommended. Importantly, amiodarone has potential severe adverse effects [70]. Prolongation of the QT interval is typical, but torsade de pointes are uncommon (<0.5%) [71]. Hypo- and hyperthyroidism are the most common extracardiac side effects of amiodarone (>20%); thus, the thyrotropin (TSH) and free thyroid hormone (fT4, fT3) levels should be checked before treatment and every six months thereafter. Photosensitivity, corneal deposits, and neurological side effects are also frequent, while pulmonary and hepatic toxicity are rare but potentially life-threatening adverse effects of amiodarone.

Digoxin inhibits the sodium-potassium pump, increasing the calcium availability to the contractile apparatus [72]. Digoxin controls the ventricular response through direct action on the atrioventricular node and by a centrally mediated vagal stimulation. Despite its efficacy in controlling resting heart rates, it is not a converter, and its benefit decreases with adrenergic stress, limiting its efficacy in critically ill patients. On the other hand, the positive inotropic effect of digoxin may be beneficial for patients with heart failure. The plasma half-life ranges from 20 to 50 hours in patients with normal kidney function and increases up to 4–6 days in patients with end-stage renal disease [72]. In addition, drug interactions may reduce digoxin clearance and electrolyte disturbances, such as hypokalemia, hypomagnesemia, and hypercalcemia, and exacerbate digoxin toxicity. In critically ill patients, for whom a rapid control of heart rate is desired, we administer 0.25 mg digoxin intravenously every 4 to 8 hours up to a cumulative dose of 1.0 to 1.5 mg, followed by a maintenance dose of 0.25 mg once daily. In patients with impaired kidney function, the maintenance dose must be reduced (0.125 mg daily for a creatinine clearance of 60–90 mL/min and 0.125 every other day for a creatinine clearance of 30–60 mL/min) [72]. To avoid adverse events, regular surveillance of electrolytes and signs of digitalis toxicity (see below) are recommended. Serum digoxin levels (measured at least 6 hours after the last dose) may be helpful to corroborate the diagnosis of toxicity but are not recommended for routine use [73]. Digoxin can cause a broad spectrum of ventricular and supraventricular arrhythmias, such as ectopic rhythms, pacemaker depression, or conduction abnormalities. Visual disturbances (blurred vision, flashing lights, halos, and green or yellow patterns), nausea, and vomiting are typical extracardiac manifestations of digoxin toxicity. Dialysis is an ineffective treatment for intoxication, but the administration of digoxin immune Fab is highly effective in life-threatening digoxin poisoning [74].

Nondihydropyridine calcium channel blockers (e.g., diltiazem, verapamil) are an alternative treatment for patients with contraindications to beta-blockers. Verapamil is more negatively inotropic than diltiazem and must be used with caution in patients with heart failure and after cardiac surgery because of the increased incidence of conduction disorders. The initial intravenous dose of diltiazem is 0.25 mg/kg over 2 min. If the response is inadequate, a second dose of 0.35 mg/kg over 2 min after 15 min or a continuous infusion of 10–15 mg/h is administered. The usual intravenous dose of verapamil is 2.5–5 mg over 2 min and may be followed by 5–10 mg after 15–30 min.

Dronedarone is an oral multichannel blocker, which compared to amiodarone has a reduced lipophilicity and no iodine components. It showed promising efficacy in multiple trials [75, 76]; however, increased mortality in patients with heart failure and risks of severe hepatotoxicity are of concern [77–80]. Dronedarone has not been evaluated in critically ill patients and is not yet available for intravenous administration, limiting its use in ICU settings.

The class Ic agents flecainide and propafenone are efficacious in restoring sinus rhythm but are associated with increased mortality in patients with structural heart disease [81]. Therefore, they cannot be generally recommended in ICU patients.

Vernakalant is a new antiarrhythmic agent that targets atrial specific channels and has been approved for pharmacological cardioversion of AF of ≤7 days duration [76, 83–85]. Vernakalant is given intravenously at an initial dose of 3 mg/kg over 10 min. If conversion fails, a second dose of 2 mg/kg is given after 15 min. Nausea, transient dysgeusia, and sneezing are common side effects. Vernakalant has also been studied after cardiac surgery, showing a conversion rate of nearly 50%, with a low incidence of severe side effects (hypotension and complete atrioventricular block) [86]. So far, data about the efficacy and safety of vernakalant in critically ill patients are lacking.

### 3.7. Long-Term Treatment after Haemodynamic Stabilisation

#### 3.7.1. Anticoagulation

AF can be complicated by thrombus formation and embolisation. Approximately 25% of ischemic strokes are caused by cardiogenic emboli, and almost half of them occur in patients with AF [28]. The risk of these complications is even higher in critically ill patients due to ongoing inflammation and a procoagulatory state [87]. Thus, all patients with AF lasting for more than 48 hours should be evaluated for anticoagulation. The scoring systems for the stratification of cardioembolic risk are the CHADS_2_ [88] and the newer CHA_2_DS_2_-VASc scores (Tables 3 and 4) [89]. Although not validated for the setting of critically illness, those scores may help clinicians in the decision about antithrombotic therapy. Patients with no risk factors are at a truly low risk and do not benefit from antithrombotic therapy [89–92]. All other patients should receive a long-term anticoagulation therapy unless they have a markedly increased bleeding risk [93]. We accomplish short-term anticoagulation by an intravenous infusion of unfractionated heparin. Without relevant bleeding, 10,000 IU per 24 hours is initiated 6 hours postoperatively and is increased in steps of 2500–5000 IE. Antifactor Xa activity is measured 6 hours after each dose adjustment (target 0.3 to 0.7 IU/mL). Alternatively the activated partial thromboplastin time (aPTT) may be used (target 1.5–2.5 times the mean of the reference range). Long-term anticoagulation with oral coumarins (e.g., warfarin, phenprocoumon, acenocoumarol) is initiated when bleeding has ceased and no invasive intervention is imminent (target INR 2.0 and 3.0) [94, 95]. After an overlapping treatment, heparin is stopped when the INR is in the target range for two days. To assess the individual bleeding risk under oral anticoagulation, the HAS-BLED score was proposed (Table 5) [96, 97], although not validated for the ICU setting. The new thrombin inhibitors (dabigatran) [98] and oral factor Xa inhibitors (rivaroxaban [99], apixaban [100, 101]) cannot be recommended in critically ill patients at the current time due to a lack of data in this particular population.

#### 3.7.2. Further Management

Up to two-thirds of patients experiencing a first episode of AF will spontaneously convert into sinus rhythm within 24 hours [102]. Thus, maintenance therapy with an antiarrhythmic drug after the first episode of AF may often be omitted or discontinued before hospital discharge [30]. An elective cardioversion is generally recommended for patients with a recent onset AF as well as in patients who remain symptomatic despite optimal rate control. After discharge from the ICU selected patients may benefit from catheter-based pulmonary vein isolation [38, 39, 41, 103], surgical treatment of AF (Cox Maze III procedure) [104], or AV node ablation with permanent (biventricular) pacing [105, 106] to reduce symptoms and increase functional performance. A multidisciplinary approach in those cases is necessary. In patients with contraindications to anticoagulation therapy, surgical or percutaneous occlusion of the left-atrial appendage is recommended [6, 107].

## 4. Conclusions

AF is the most common arrhythmia in the ICU, and it can precipitate hypotension and heart failure. Despite recent advances in the field and new published guidelines, the therapeutic armamentarium for ICU patients remains limited. ECV is the treatment of choice for patients with severe symptoms, but its efficacy is limited. Amiodarone, beta-blockers, calcium channel blockers, and digoxin are used most frequently, but their use is often complicated by adverse effects. Newer drugs, such as dronedarone and vernakalant, have not been generally introduced into the ICU setting yet because they are not available intravenously, are contraindicated with structural heart disease, or are disadvised due to haemodynamic instability. New substances with high efficacy, favourable haemodynamic effects, and a low risk profile are urgently needed.

## Figures and Tables

**Table 1:** Modifiable promoters of atrial fibrillation.

Mechanism	Etiology	Specific treatment options
Myocardial stretch (atrial hypertension, atrial dilatation, reduced contractility)	Fluid overload Acute mitral insufficiency Mitral stenosis	Fluid removal (restrictive fluid administration, diuretics, renal replacement therapy) Intra-aortic balloon pump; cardiac surgery Valvuloplasty

Inappropriate oxygen delivery to the myocardium	Myocardial ischemia Hypovolemia Anemia	Revascularization Fluid challenge Transfusion of red blood cells

Electrolyte disturbances (risk factors: diuretics, dialysis)	Hypokalemia Hypomagnesemia	Substitution of potassium (goal K^+^ 4.5–5.5 mmol/L) Substitution of magnesium (goal Mg^++^ > 1.0 mmol/L)

Systemic and local inflammation	Heart-lung machine Sepsis Myocarditis	Steroids; off-pump cardiosurgical techniques Antimicrobial therapy Immunosuppression

Adrenergic overstimulation	Inotropic support Stress (pain, anxiety)	Reduction of inotropes Sedation; analgesia; betablockers

Endocrine disorder	Elevated thyroid hormones Pheochromocytoma	Betablockers; thyreostatic drugs Alpha- and betablockers

Various	Hypothermia	Correction of hypothermia

**Table 2:** Frequently used intravenous antiarrhythmic substances in the ICU.

Substance	Dosing	Half-life	Commentary
Esmolol	1.0 mg/kg in boluses of 10–20 mg iv, followed by continuous infusion (start with 0.05 mg/kg/min, increase dose every 30 minutes if necessary)	7–10 min	Good efficacy in high adrenergic state. Positive effect on cardiovascular comorbidities. Consider negative inotropic effects

Diltiazem	0.25 mg/kg iv over 2 minutes, followed by continuous infusion (10–15 mg/h) if necessary	2–4 h	Longer half-life as esmolol. No beta-blocking effects. Consider negative inotropic effects

Amiodarone	150–300 mg iv, followed by a continuous infusion (900–1200 mg daily) up to 0.1 g/kg Maintenance dose 200 mg daily	20–100 d	Good efficacy, safe in patients with structural heart disease. Extreme long half-life up to 80 days. Consider extracardiac side effects

Digoxin	0.25–0.5 mg iv every 4–8 h up to 1 mg, followed by maintenance dose of 0.25 mg daily	20 h–6 d	Positive inotropic effect. Reduce dose in renal dysfunction. Check digoxin plasma levels to avoid toxicity

**Table 3:** The CHA2DS2-VASc score: pointing system (modified from [82]).

Risk factors	Points
Chronic heart failure	1 pt
Hypertension	1 pt
Age 65–74 years	1 pt
Age >75 years	2 pts
Diabetes mellitus	1 pt
Stroke/TIA	2 pts
Vascular disease	1 pt
Sex (female)	1 pt

Legend: TIA: transient ischemic attack.

**Table 4:** The CHA2DS2-VASc score: theoretical risk of stroke/thromboembolism per patient year without anticoagulation (modified from [82]).

Score	Yearly TE rate
0	0%
1	1.3%
2	2.2%
3	3.2%
4	4.0%
5	6.7%
6	9.8%
7	9.6%
8	6.7%
9	15.2%

Legend: TE: thromboembolism.

**Table 5:** The HAS-BLED score: pointing system.

Risk factors	Points
Hypertension	1 pt
Abnormal renal/liver function	1 pt each
Stroke	1 pt
Bleeding (prior)	1 pt
Labile INR	1 pt
Elderly (age >65 years)	1 pt
Drugs and alcohol	1 pt each

**Score **≥**3 indicates high risk of major bleeding (>3.7%/y)**	

Legend: INR international normalized ratio.

## Abbreviations

AF: Atrial fibrillation
ECV: Electrical cardioversion
ECG: Electrocardiogram
ICD: Internal cardioverter/defibrillator
INR: International normalised ratio
ICU: Intensive care unit
TSH: Thyroid-stimulating hormone (thyrotropin).
